# ComiRNet: a web-based system for the analysis of miRNA-gene regulatory networks

**DOI:** 10.1186/1471-2105-16-S9-S7

**Published:** 2015-06-01

**Authors:** Gianvito Pio, Michelangelo Ceci, Donato Malerba, Domenica D'Elia

**Affiliations:** 1 Department of Computer Science, University of Bari Aldo Moro, Via Orabona, 4, I-70125 Bari, Italy; 2 CNR, Institute for Biomedical Technologies, Via Amendola 122/D, I-70126 Bari, Italy

**Keywords:** miRNA-gene regulatory networks, Semi-supervised learning, Biclustering, miRNA database

## Abstract

**Background:**

The understanding of mechanisms and functions of microRNAs (miRNAs) is fundamental for the study of many biological processes and for the elucidation of the pathogenesis of many human diseases. Technological advances represented by high-throughput technologies, such as microarray and next-generation sequencing, have significantly aided miRNA research in the last decade. Nevertheless, the identification of true miRNA targets and the complete elucidation of the rules governing their functional targeting remain nebulous. Computational tools have been proven to be fundamental for guiding experimental validations for the discovery of new miRNAs, for the identification of their targets and for the elucidation of their regulatory mechanisms.

**Description:**

ComiRNet (Co-clustered miRNA Regulatory Networks) is a web-based database specifically designed to provide biologists and clinicians with user-friendly and effective tools for the study of miRNA-gene target interaction data and for the discovery of miRNA functions and mechanisms. Data in ComiRNet are produced by a combined computational approach based on: 1) a semi-supervised ensemble-based classifier, which learns to combine miRNA-gene target interactions (MTIs) from several prediction algorithms, and 2) the biclustering algorithm HOCCLUS2, which exploits the large set of produced predictions, with the associated probabilities, to identify overlapping and hierarchically organized biclusters that represent miRNA-gene regulatory networks (MGRNs).

**Conclusions:**

ComiRNet represents a valuable resource for elucidating the miRNAs' role in complex biological processes by exploiting data on their putative function in the context of MGRNs. ComiRnet currently stores about 5 million predicted MTIs between 934 human miRNAs and 30,875 mRNAs, as well as 15 bicluster hierarchies, each of which represents MGRNs at different levels of granularity. The database can be freely accessed at: http://comirnet.di.uniba.it.

## Background

Deciphering the modular organization of gene regulatory networks is crucial for the understanding of biological processes at a system-wide level [1]. MicroRNAs (miRNAs) represent the largest class of small non-coding RNAs (20-24 nucleotide long (nt)), acting as post-transcriptional regulators of gene expression in viruses, plants and animals. Since their discovery in 1993 [2], the number of scientific reports aiming to elucidate their structural and functional properties has been growing at an impressive rate because of the discovery of their pivotal role in important biological processes, in almost all organisms and in a large number of human diseases [3]. This is also reflected in the huge amount of computational approaches, models and tools that have been proposed with the aim of identifying new miRNA genes and targets and of elucidating regulatory mechanisms through which they are able to fine-tune gene expression.

Computational approaches are indeed fundamental for both gene-specific and large-scale predictions of miRNA targets, for the formulation of new functional hypotheses and for guiding experimental validations. In a general classification of computational approaches, which are typically developed in the research concerning miRNAs, we can substantially distinguish two main categories: *i) *algorithms and tools for miRNA target site predictions and *ii) *integrated tools for the discovery of miRNA-gene target interaction networks, based on the combinatorial regulatory properties of miRNAs and the exploitation of gene expression data.

Concerning *i)*, since miRNA targeting is guided by sequence complementarity [4], almost all the prediction algorithms developed so far are primarily based on pairing rules and evolutionary sequence conservation. Several algorithms have been used to generate miRNA target databases, such as MicroCosm (based on the predictions of the miRanda algorithm [5]), TargetScan [6], PicTar [7] and Diana-microT [8]. Other resources, such as mirDIP [9] and TarMiR (http://www.tarmir.rgcb.res.in), integrate pre-computed miRNA targets from several commonly used miRNA target prediction databases and provide tools for a comprehensive and customizable search.

miRTar [10] and EIMMo [11], in addition to target predictions, provide tools for the enrichment analysis of targeted genes in pathways. However, their effectiveness is negatively affected by high uncertainty and by the extreme complexity of rules governing miRNA functional targeting, whose mechanisms still remain elusive [12]. In particular, miRNAs act as repressors or inhibitors of mRNA translation by adopting several mechanisms. In animals, an important aspect for target recognition is a short sequence (6-8 nt long), the so-called "seed region", that matches the target, generally located in the 3' untranslated regions (3' UTRs) of messenger RNAs (mRNAs). Transcript degradation or translational repression is induced, depending on perfect or imperfect seed pairing, respectively [13]. In mammals, besides seed pairing (6-8 nt long), five general features of the site context seem to significantly contribute to boost site efficacy [14]. A study by Wang-Xia et al. [15], suggests that individual miRNAs can have distinct sequence determinants that lead to mRNA targeting, some miRNAs having target sequences in the 3' UTR of mRNAs and others in the mRNA coding sequence (CDS), but not in the 3' UTR. More recent works confirm that miRNA target sites located in the CDS can effectively inhibit translation [16]. Others speculate that the key mechanism through which miRNAs are able to flexibly tune the time scale and the magnitude of their post-transcriptional regulatory effects may be precisely the combination of CDS and UTR target site binding of miRNAs [17]. Finally, a further element of complexity has arisen from recent experiments that have consistently revealed extensive AGO-associated mRNAs that lack seed complementarity with miRNAs. This study reveals a novel function of AGOs: the central catalytic component of the RNA-induced silencing complex (RISC), as sequence-specific RNA-binding proteins, which may aid miRNAs in recognizing their targets with a high specificity [18]. Thus, a target prediction model should also take into account the influence of AGO target sites.

As new structural and functional features of miRNA targeting are discovered, new tools are developed aiming to provide predictions that are as effective as possible, but the picture that currently emerges is so complex that it would be unrealistic to think of solving the problem by using only one algorithm, approach or model. Some encouraging results have been reported for methods which combine different prediction algorithms [19], however, they are still preliminary.

As regards *ii)*, the availability of a huge amount of expression data produced by high-throughput technologies, such as microarrays and next-generation sequencing (NGS), has led to the development of several computational models and tools able to infer miRNA-gene target regulatory networks, by using inverse correlation measurements and targeting predictions [20-23]. Some of these tools combine sequence-specific target identification, by using predictions from only one predictive algorithm, while others consider the combination of more algorithms (usually 3 to 5). Moreover, disease association, genomic annotation and the integration of information extracted from the literature facilitate the functional investigation of miRNAs. mirConnX [22] integrates computationally-predicted transcription factor (TF)-gene associations with the miRNA target predictions. Dynamic TF-and miRNA-gene associations are inferred from user-provided expression data, using a chosen association measure.

However, such types of tools strongly rely on the relative miRNA expression levels, which, as is well-known, strongly depend on the biological conditions of the samples used. Furthermore, even though it is possible to determine miRNA targets by examining significant inverse correlation between miRNA and mRNA expression data, this cannot be applied as a general rule for miRNA target identification, since miRNA targeting does not necessarily lead to mRNA degradation. In order to be effective, a computational tool aiming to identify miRNA-gene target regulatory networks (MGRNs) by using expression data should include proteomic data, providing a measure of the corresponding levels of proteins. Nevertheless, this would not exclude the prediction bias due to the specific expression properties of miRNAs, which are space- and time-related with respect to the biological processes they are able to control.

In this paper we present ComiRNet, a user-friendly web-based system that has been developed for the efficient query, retrieval, export, visualization and analysis of predicted miRNA-gene target interactions (MTIs) and MGRNs. Data in ComiRNet are produced by a combined computational approach based on: 1) a semi-supervised ensemble-based classifier [24], which *learns to combine *MTIs from several prediction algorithms and 2) the biclustering algorithm HOCCLUS2 [25], which exploits the large set of produced predictions, with the associated probabilities, to identify overlapping and hierarchically organized biclusters (i.e., MGRNs).

The ComiRNet database stores about 5 million predicted interactions between 934 human miRNAs and 30,875 mRNAs, and 15 bicluster hierarchies, identified by HOCCLUS2 with different values of its parameters.

It distinguishes from other databases by the following innovative aspects:

• the predictions of MTIs are based on combinations of several algorithms, since this approach provably outperforms, both in precision and recall, methods based on single predictive models [24];

• the proposed combination does not rely on simple averaging approaches, which have been demonstrated to be less effective when applied on large-scale prediction data sets (see [24,25]).

• miRNA-mRNA interactions are identified by means of a semi-supervised ensemble learning approach, which is able to increase the reliability of predictions and to help in inferring significant and functional related MGRNs [24], by exploiting both validated and predicted interactions;

• active concurrency of miRNAs and mRNAs (i.e., expression data) is purposely neglected to avoid a bias in data construction, due to the specific context of the system analyzed;

• contrary to similar methods, mainly based on the assumption of finding reasonable biological solutions by incorporating prior knowledge [26], no additional knowledge is required. This increases the possibility to explore as many biological scenarios as possible.

As regards the functionalities, when compared with other similar web-based tools, ComiRNet offers biologists and clinicians a unique tool to easily:

• explore, without any prior knowledge and functional information, all the possible cooperative interactions that a miRNA can potentially establish with other miRNAs on specific groups of genes;

• explore and analyze the extent to which a group of biclustered miRNAs (i.e., the miRNA module) can influence a group of functionally-related target genes (i.e., the gene module);

• explore the extent to which some miRNAs can participate in alternative and related modules (i.e., MGRNs) and hence elucidate miRNA overlapping or concurrent functions in specific pathways or biological processes;

• discover new functional miRNA targets and, thus, new functional properties of miRNAs.

## Construction and content

ComiRNet is a web application built by exploiting the Play 2.2 framework (http://playframework.com), which runs on the application server Netty (netty.io). Data are stored in a PostgreSQL relational database and include: gene symbols, miRNA IDs, the predicted interactions with the associated score and a set of hierarchically organized and overlapping MGRNs.

Moreover, stored data are integrated with external resources, namely:

• miRBase [5], for miRNA information. The association is made through miRNA IDs;

• GeneCards [27] and Entrez Gene [28], for gene information. For the former, the association is made through gene symbols, while for the latter the association is performed through Entrez IDs provided by NCBI;

• miRTarBase [29], for information about the experimental validation of each interaction. The integration is performed by associating each miRNA-gene pair to the corresponding miRTarBase accession id.

Notably, miRBase can be queried on the basis of miRNA IDs, while GeneCards can be queried on the basis of gene symbols. This allows ComiRNet to automatically generate links to external resources which are safe from possible updating problems. On the contrary, both miRTarBase and Entrez Gene use proprietary IDs for interactions and genes, respectively. To ensure a correct mapping, ComiRNet must use these proprietary IDs which do not change over time. Therefore, newly inserted interactions and genes are periodically added by the system through a procedure which retrieves new entries from the external resources.

In the following subsections we report a brief summary of the methods adopted to construct data stored in ComiRNet. In particular, we describe the semi-supervised ensemble-based learning method [24], used to combine the output of several prediction algorithms, and the bicluster algorithm HOCCLUS2 [25], adopted to identify MGRNs, as well as their tight integration. As we will clarify during the paper, this integration is fully exploited in ComiRNet.

### Learning to combine predictions

The considered task of learning to combine several prediction algorithms raises some issues that must be taken into account and that make classical machine learning approaches inappropriate. In particular: *i) *very few interactions are experimentally validated and can be considered "stable" examples; *ii) *only positive examples of interactions are available, whereas negative examples are not generally available and, when available, their number is small; *iii) *prediction algorithms consider similar features and their combination can lead to collinearity problems [30].

In order to face *i) *and *ii)*, we adopted the semi-supervised learning algorithm proposed in [31], which considers both positively labeled examples of interactions and the huge set of available unlabeled (unknown) instances. As for *iii)*, the collinearity problem is alleviated by considering as features the scores (outputs) obtained by the prediction algorithms (instead of original features), resorting to a solution similar to meta-learning algorithms. The advantage of applying machine learning techniques to the outputs of prediction algorithms consists in automatically adapting to unknown patterns of the outputs and performing more reliable predictions when these patterns occur [24].

The proposed method consists of three main steps:

1 Representation: each example of interaction is represented by a vector of scores, obtained by prediction algorithms, and is associated with a label representing the fact that it is experimentally validated (positive label) or not (unlabeled).

2 Learning of a non-traditional classifier: a probabilistic classifier is learned to compute the likelihood that an example of interaction is positively labeled (known) / unlabeled.

3 Learning of a weight-aware classifier: a new probabilistic classifier which also exploits the likelihood computed in the step *2) *is learned. This classifier associates a score with each interaction to decide whether this interaction is true.

In step *3)*, scores are computed by assuming that all the labeled examples are randomly sampled from the set of all positive examples. Thus, the probability that an existing interaction belongs to the set of labeled examples is independent of the specific interaction. Formal definitions can be found in [24].

The learning tasks in steps *2) *and *3) *are challenged by the highly unbalanced training data. Indeed, both the labeled examples used in step *2) *and the positive examples used in step *3)*, are significantly few in number compared to the size of the training data sets. This motivates the development of an ensemble-based approach. In particular, *K *classifiers are learned from all positive examples and a subset of negative examples, randomly sampled with replacement. The score associated with each example is computed by averaging the output of all the classifiers that considered that specific example during the learning phase. Details on this ensemble-based approach are reported in [24].

### Discovering miRNA:mRNA regulatory networks

The regulatory networks stored in ComiRNet are extracted by applying the biclustering algorithm HOCCLUS2 [25] to the set of predicted interactions, identified with the method described in the previous section. HOCCLUS2 extracts highly-cohesive, possibly overlapping and hierarchically organized biclusters in three main steps.

In the **first step**, HOCCLUS2 builds biclusters in the form of bicliques by analyzing interactions in both directions, from miRNA to mRNA and from mRNA to miRNA. The only input parameter is *β *∈ [0, 1], the minimum score to consider a miRNA:mRNA interaction as "reliable." Once a set of bicliques is obtained for each direction, they are merged to obtain a single set of bicliques.

Here we describe the extraction of bicliques in the "miRNA to mRNA" direction. HOCCLUS2 takes into account the following statistics:

• *avg mirna*, i.e. the average number of miRNAs which target each mRNA with a score greater than *β*;

• *abs_min_mrna *and *min_mrna*, i.e. the *absolute *and the *outlier-proof *minimum number of mRNAs which are targeted by each miRNA with a score greater than *β*.

The value of *min_mrna *is computed by discarding the lowest 0.15% values (possibly outliers, according to the 3*σ *rule), assuming that the number of mRNAs which are targeted by each miRNA follows a Gaussian distribution.

**Figure 1:**
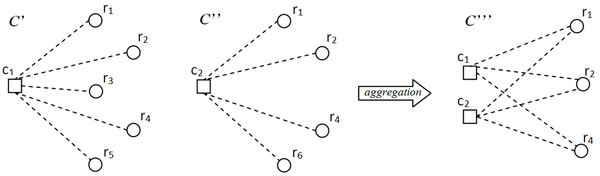
**Example of an aggregation of two biclusters **$C^{\prime }$ and $C^{\prime \prime }$.This Figure is taken from [25].

Once these statistics are computed, an initial set of bicliques is built, where each biclique consists of a single miRNA and the set of mRNAs it targets with a score greater than *β*. Then, the algorithm iteratively aggregates two bicliques $C^{\prime }$ and $C^{\prime \prime }$ into a new biclique $C^{\prime \prime \prime }$ as follows: $C_{r}^{\prime \prime \prime }=C_{r}^{\prime }\cap C_{r}^{\prime \prime }$; $C_{c}^{\prime \prime \prime }=C_{c}^{\prime }\cup C_{c}^{\prime \prime }$ (see Figure 1), where *C_r _*and *C_c _*are the sets of mRNAs and miRNAs in *C*, respectively. The necessary condition for bicliques aggregation is:

$C_{r}^{\prime }\cup C_{r}^{\prime \prime }$ ≥ *min mrna *and $C_{c}^{\prime \prime \prime }=C_{c}^{\prime }\cap C_{c}^{\prime \prime }$*≤ avg mirna*

since a good biclique should approximately contain *avg_mirna *miRNAs, while keeping the highest possible number of mRNAs (at least *min_mrna*). In addition, highly cohesive bicliques are desirable, therefore, among the possible aggregations of pairs of bicliques $\left\langle C^{\prime },C^{\prime \prime }\right\rangle$, we select the one for which the following measure is maximized:

*jaccard $\left({C^{\prime }}_{r},{C^{\prime \prime }}_{r}\right)$ ∗ q*(*aggregate $\left(C^{\prime },C^{\prime \prime }\right)$, A*)

where *jaccard $\left({C^{\prime }}_{r},{C^{\prime \prime }}_{r}\right)=\frac{\left|{C^{\prime }}_{r}\cap {C^{\prime \prime }}_{r}\right|}{\left|{C^{\prime }}_{r}\cup {C^{\prime \prime }}_{r}\right|}$*, *q*(⋅,⋅) is a cohesiveness function and A is the adjacency matrix containing the score associated with each interaction. In [25] we defined *q *as $q\left(C,A\right)={\left(\left|C_{r}\right|*\left|C_{c}\right|\right)}^{-1}*\underset{x\in C_{r}}{\sum }\underset{y\in C_{c}}{\sum }A_{x,y}$, which measures the weighted (i.e. by considering the scores) percentage of interactions in a biclique, normalized by the maximum number of possible interactions. Since this measure is also known as *compactness*, henceforth, both the terms *compactness *and *cohesiveness *will be used to refer to the same concept.

Finally, in this first step, bicliques containing less than *abs_min_mirna *miRNAs or less than *abs_min_mrna *mRNAs are pruned. Objects which do not belong to any biclique are considered isolated/noise objects.

In the **second step**, overlapping biclusters are iteratively identified and merged. We assume that that two non-overlapping biclusters should be separable in the space. Thus, given two biclusters $C^{\prime }$ and $C^{\prime \prime }$ belonging to the same hierarchical level, we identify two optimal separating hyperplanes between $C^{\prime }$ and $C^{\prime \prime }$ by learning an SVM model for each dimension (miRNAs and mRNAs). Objects in $C^{\prime }$ and $C^{\prime \prime }$ are used as both training set and testing set. Misclassified objects are those which possibly belong to both biclusters and are added to the bicluster which previously did not contain them. In this way, overlapping biclusters are identified.

As regards biclusters merging, we assume that miRNAs and mRNAs are normally distributed and consider the distance between pairs of biclusters. Two biclusters $C^{\prime }$, $C^{\prime \prime }$ are candidates for merging if they are close according to at least one dimension, that is:

$dist\left({C^{\prime }}_{r},{C^{\prime \prime }}_{r}\right)-2\sigma \left({C^{\prime }}_{r}\right)-2\sigma \left({C^{\prime \prime }}_{r}\right)\leq 0$ or $dist\left({C^{\prime }}_{c},{C^{\prime \prime }}_{c}\right)-2\sigma \left({C^{\prime }}_{c}\right)-2\sigma \left({C^{\prime \prime }}_{c}\right)\leq 0$ where *dist*(*w, z*) is the Euclidean distance between the centroids of the clusters *w *and *z*, and *σ*(*w*) is the standard deviation of the cluster *w*. Merging occurs, only if the quality constraint $q\left(C^{\prime \prime \prime },A\right)>\alpha$ is satisfied, where $C_{r}^{\prime \prime \prime }\leftarrow C_{r}^{\prime }\cup C_{r}^{\prime \prime }$,$C_{c}^{\prime \prime \prime }\leftarrow C_{c}^{\prime }\cup C_{c}^{\prime \prime }$ and *α *is a user-defined threshold. Low values of *α *facilitate merging, decreasing cohesiveness. Since a bicluster can be a candidate for multiple merging, we only perform the one resulting in the bicluster with maximum cohesiveness. Further details about the merging phase can be found in [25].

In the **third step **biclusters are ranked according to a preference function based on the *p*-value of a statistical test for the following hypothesis: the mRNAs which belong to a specific bicluster are, on average, more functionally similar to other mRNAs in the same bicluster than to mRNAs which belong to other biclusters. The functional similarity between two genes is evaluated by means of the *SimGIC *measure [32], computed according to the genes' annotations in Gene Ontology (GO).

*SimGIC *is defined as follows:

$$SimGIC\left(x_{1},x_{2}\right)=\frac{\underset{t\in GO\left(x_{1}\right)\cap GO\left(x_{2}\right)}{\sum }IC\left(t\right)}{\underset{t\in GO\left(x_{1}\right)\cup GO\left(x_{2}\right)}{\sum }IC\left(t\right)},$$

where *GO*(*x*) represents the set of GO terms which *x *is associated with, and *IC*(*t*) = *− *log *p*(*t*) is the negative log-likelihood of the term *t *computed on the basis of the prior probability *p*(*t*) of *t*. *p*(*t*) is estimated as the percentage of genes associated with the term *t*, according to the UniProt Homo sapiens GO annotations.

The statistical test we consider is the classical one-tailed Student's *t *test that allows us to evaluate the null hypothesis *H*_0 _: *µ*_0_(*C*) = *µ*(*L, C*) against *H*_1 _: *µ*_0_(*C*) *>µ*(*L, C*), where *µ*_0_(*C*) is the mean of the intra-bicluster functional similarities of *C *and *µ*(*L, C*) is the mean of the inter-bicluster functional similarities between the bicluster *C *and the other biclusters belonging to the same hierarchy level of *C*. Details of the formal definition of *µ*_0_(*C*) and *µ*(*L, C*) can be found in [25].

The lower the *p*-value, the higher the difference between the average intra-functional similarity and the average inter-functional similarity and the better the rank of the bicluster. Since we compute SimGIC according to two different hierarchies of GO, i.e. Molecular Function (MF) and Biological Process (BP), we compute two different rankings on the basis of two different *p*-values, i.e. *p_BP _*and *p_MF _*.

### Significance of the extracted MGRNs

The combination in ComiRNet of the proposed approaches for learning to combine predictions and for extracting miRNA-gene networks leads to the best results in terms of biological significance, when compared with other baseline combination strategies. As baseline combination strategies we considered:

• *Score averaging - three best *(**SA-3B**): an algorithm that equally weights the contribution of the best three prediction algorithms (TargetScan Conserved, PITA Top Hits and picTar 5-way), according to [33]. In this case, we used HOCCLUS2, METIS [34] and ROCC [35] as biclustering algorithms.

• *Weighted score averaging - three best *(**WSA-3B**): an algorithm that weights the contribution of the best three prediction algorithms (TargetScan Conserved, PITA Top Hits and picTar 5-way). The weights are proportional to the reliability (computed on the basis of the F-Score) of each algorithm, according to [33]. In this case, we used HOCCLUS2, METIS [34] and ROCC [35] as biclustering algorithms.

• *Score averaging *(**SA**): a simple algorithm that equally weights the contribution of each single prediction algorithm. In this case, we used HOCCLUS2 as biclustering algorithm.

It is noteworthy that METIS requires as input the number of biclusters to extract and that it identifies a single set of biclusters (not hierarchically organized). Thus, for fair comparison, METIS is required to return the same number of biclusters returned by HOCCLUS2 at the first level of the hierarchy.

In Table 1, we report a summary of the obtained results. Focusing on the prediction of MTIs, it is possible to see that the proposed approach always lets HOCCLUS2 identify at least one hierarchy level with very low *p_BP _*and *p_MF _*values, independently of the choice of its parameters. Moreover, comparing the results with those obtained with the SA approach (which is the best among the considered competitors), it is noteworthy that a smaller number of biclusters is extracted, grouping less miRNAs and mRNAs. This is due to the fact that the solution we adopt in ComiRNet to identify MTIs is able to better filter out false positives and allows HOCCLUS2 to focus only on more reliable interactions.

**Table 1:** Quality of biclusters obtained by different combination strategies.

*α*	*β*	*N *			best level		
		**(mRNA/miRNA)**	**lev**	**#cc**	***p_MF_***	***p_BP_***	***µ_q_***

**SA-3B + METIS**							

-	-		1	700	1.000	1.000	0.36
-	-	13714/703	1	619	1.000	1.000	0.49
-	-		1	599	1.000	1.000	0.35

**SA-3B + ROCC**							

-	-	101/9	1	122	1.000	1.000	0.01

**SA-3B + HOCCLUS2**							

0.1	0.3		9	350	0.000	0.000	0.41
0.2	0.3	5698/612	7	210	0.000	0.000	0.31
0.3	0.3		5	700	1.000	1.000	0.49

0.1	0.4		8	155	0.004	0.009	0.32
0.2	0.4	4735/607	7	144	0.006	0.001	0.24
0.3	0.4		6	619	1.000	1.000	0.52

0.1	0.5		8	77	0.345	0.167	0.27
0.2	0.5	3337/572	7	108	0.257	0.112	0.26
0.3	0.5		6	205	1.000	0.206	0.35

**WSA-3B + METIS**							

-	-	13714/703	1	758	1.000	1.000	0.29
-	-		1	667	1.000	1.000	0.39
-	-		1	622	1.000	1.000	0.35

**WSA-3B + ROCC**							

-	-	101/9	1	122	1.000	1.000	0.01

**WSA-3B + HOCCLUS2**							

0.1	0.3		9	379	0.000	0.000	0.41
0.2	0.3	6209/618	7	221	0.001	0.000	0.31
0.3	0.3		6	758	1.000	1.000	0.50

0.1	0.4		7	58	0.094	0.016	0.21
0.2	0.4	5122/601	6	148	0.053	0.004	0.25
0.3	0.4		5	667	1.000	1.000	0.54

0.1	0.5		8	156	0.151	0.263	0.37
0.2	0.5	3653/570	7	168	0.123	0.298	0.38
0.3	0.5		6	314	0.256	1.000	0.50

**SA + HOCCLUS2**							

0.2	0.3	8723/599	7	294	0.140	0.080	0.43
0.3	0.3		5	294	0.140	0.080	0.43

0.2	0.4	7772/620	9	416	0.008	0.000	0.33
0.3	0.4		7	830	0.000	0.000	0.42

0.2	0.5	4336/627	9	148	0.286	0.261	0.31
0.3	0.5		7	522	1.000	0.228	0.47

**ComiRNet**							

0.1	0.3		8	444	0.000	0.000	0.52
0.2	0.3	2379/614	7	444	0.000	0.000	0.52
0.3	0.3		6	309	0.000	0.000	0.42

0.1	0.4		8	148	0.000	0.000	0.39
0.2	0.4	1626/544	7	152	0.000	0.000	0.39
0.3	0.4		6	298	0.000	0.001	0.57

0.1	0.5		8	105	0.000	0.000	0.43
0.2	0.5	1245/467	7	105	0.000	0.000	0.43
0.3	0.5		7	110	0.000	0.000	0.42

*N *represents the number of biclustered mRNAs and miRNAs. *lev *represents the number of hierarchy levels. #cc is the number of biclusters. *µ_q _*is the average compactness. The "best" level is the hierarchy level with the lowest $\frac{p_{MF}+p_{BP}}{2}$ value.

On the other hand, the quality of biclusters identified by competitive biclustering algorithms is significantly lower than that obtained by HOCCLUS2. In particular, although METIS is able to obtain relatively high values of cohesiveness (*µ_q _*), the extracted biclusters appear not to be biologically coherent, in terms of *p_BP _*and *p_MF _*. The algorithm ROCC, if compared to HOCCLUS2, has poor performances in terms of all the considered quality measures, with both SA-3B and WSA-3B settings.

On the overall, by observing Table 1 it is possible to conclude that the proposed combination strategy for MTI prediction, which relies on a semi-supervised ensemble-based approach, and for MGRN identification, as implemented by the biclustering algorithm HOCCLUS2, leads to the best results, in terms of all the considered quality measures. Additional considerations from the biological view- point will be provided in the Section "Discussion".

## Utility

ComiRNet is a user-friendly web-based system for querying, retrieving and displaying predicted miRNA-gene regulatory networks (MGRNs), i.e. biclusters, and miRNA-gene target interactions (MTIs). The web interface consists of three main functions: i) querying and browsing MTIs; ii) querying MGRNs; iii) exploring and browsing MGRNs. For querying purposes, a list of gene symbols and/or miRNAs IDs can be specified as input. Results can be displayed in a table or exported as a text file, which can be used to select the MGRNs or MTIs of interest. ComiRNet enables the exploration and browsing of MGRNs through a graph-based visualization of the networks and of related super-and sub-networks (i.e., parent and child biclusters, according to the hierarchies identified by HOCCLUS2). In the following subsections we will describe these functions in detail.

### Querying and browsing MTIs

The "Search Interaction" function exploits the database of interactions obtained by the application of the semi-supervised ensemble learning algorithm described before (henceforth called "ComiRNet classifier"). The graphic interface allows users to search for MTIs associated with a list of target genes (specified by official gene symbols) or to a list of miRNAs (specified by miRNAs IDs).

In particular, by supplying a (list of) gene symbol(s) to the search form, the system returns the list of all miRNAs that are predicted to target that gene(s). Alternatively, a (list of) miRNA ID(s) can be entered, in order to view the list of all target genes of that miRNA(s). These search criteria can be used separately or in combination. The system logically combines the query conditions in AND, i.e. only those interactions satisfying both the search criteria are returned. However, it is possible to perform the query with the OR condition, in case the user is interested in this condition. Moreover, the search can be customized by applying a filter on the score (i.e. reliability) of the interactions returned by the ComiRNet classifier, whose range is in the interval [0, 1].

The results obtained by the query system are shown in a table containing the list of MTIs retrieved, according to the specified search criteria. This report includes the miRNA ID, the gene official symbol, the corresponding Entrez Gene ID, the interaction score and information on the validation of the MTI. Additional information is provided as hyperlinks, including miRNA information from miRBase [5], gene information from GeneCards [27] and Entrez Gene [28], and information about the experimental validation of the interactions from miRTarBase [29].

**Figure 2:**
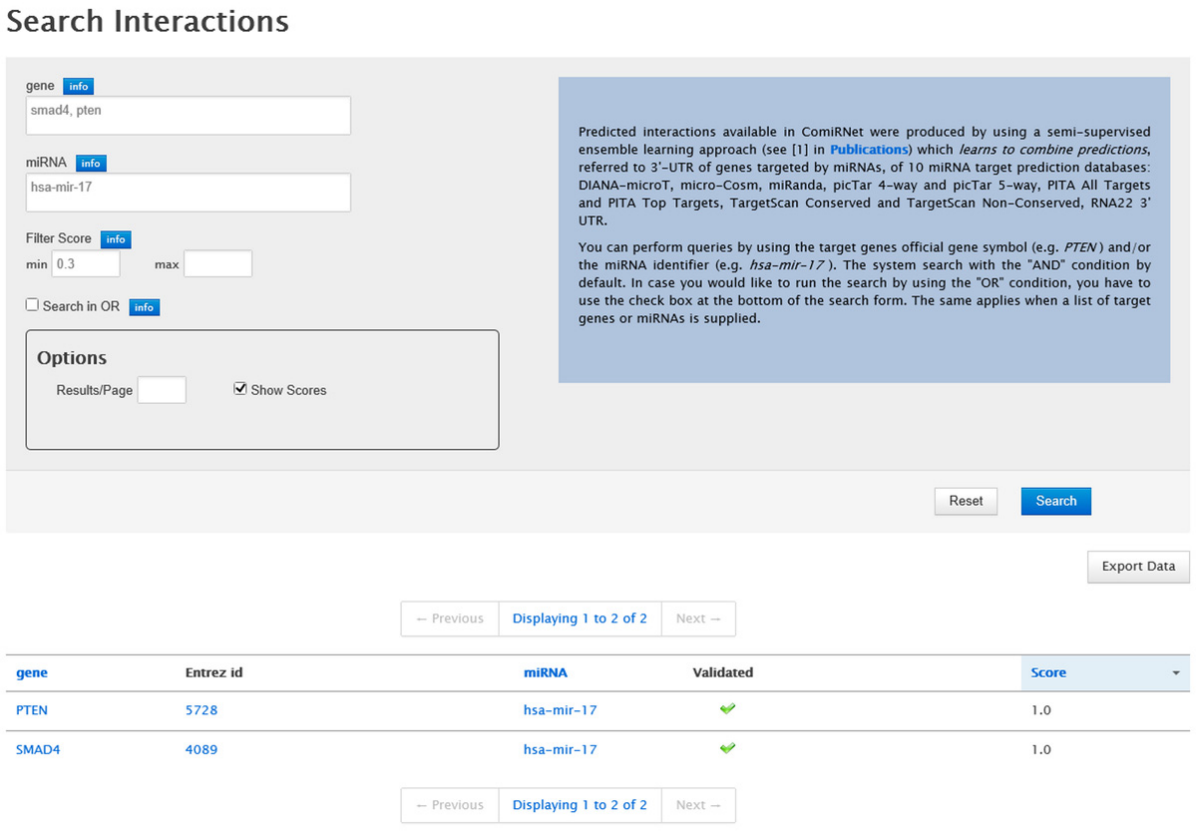
**ComiRNet MTI search form**. Results obtained by searching for MTIs involving the genes SMAD4 and PTEN and the miRNA hsa-mir-17.

The results in the table can be sorted according to the miRNA ID, the gene symbol and the interaction score. They can also be exported as a tab-delimited text file. A complete view of the search form and of the query report is provided in Figure 2.

### Querying MGRNs

MGRNs stored in the ComiRNet database have been extracted by HOCCLUS2, by exploiting MTIs identified by the ComiRNet classifier. Currently, the database stores 15 different hierarchies of MGRNs, obtained with different combinations of the parameters *α *and *β*. In Table 2 some statistics about biclusters are reported, showing the possible influence of the considered hierarchy in the number and in the quality of identified networks. In particular, Table 2 shows, for each combination of *α *and *β*, the number of hierarchy levels, the number of biclusters and the number/percentage of significant (i.e. with a *p*-value *≤ *0.05) biclusters, according to the statistical test adopted in the ranking phase of HOCCLUS2.

**Table 2:** Quantitative information on the hierarchies of MGRNs stored in ComiRNet

HIERACHY			#levels	#biclusters	#biclusters	#biclusters
**id**	***α***	***β***			***p_BP _<*0.05**	***p_MF _<*0.05**

1	0.1	0.3	8	1861	576 (30.95%)	515 (27.67%)
2	0.1	0.4	8	1229	377 (30.67%)	349 (28.39%)
3	0.1	0.5	8	866	309 (35.68%)	260 (30.02%)

4	0.2	0.3	7	2172	654 (30.11%)	639 (29.41%)
5	0.2	0.4	7	1399	443 (31.66%)	408 (29.16%)
6	0.2	0.5	7	966	350 (36.23%)	287 (29.71%)

7	0.3	0.3	6	2469	755 (30.57%)	674 (27.29%)
8	0.3	0.4	6	1570	485 (30.89%)	459 (29.23%)
9	0.3	0.5	7	1181	425 (35.98%)	398 (33.70%)

10	0.4	0.3	6	3115	873 (28.02%)	787 (25.26%)
11	0.4	0.4	6	1863	608 (32.63%)	532 (28.55%)
12	0.4	0.5	7	1371	494 (36.03%)	444 (32.38%)

13	0.5	0.3	5	3415	851 (24.91%)	735 (21.52%)
14	0.5	0.4	5	2039	623 (30.55%)	541 (26.53%)
15	0.5	0.5	5	1329	453 (34.08%)	391 (29.42%)

It is noteworthy that *α *implicitly influences the number of hierarchy levels and the number of biclusters at each hierarchy level. In particular, the higher the value of *α*, the lower the number of hierarchy levels and the higher the number of biclusters per level. From a biological viewpoint, higher values of *α *lead to smaller biclusters, giving more emphasis to pathway-specific interactions, with respect to inter-pathway connections.

Moreover, higher values of *β *lead to two consequences: first, a smaller number of biclusters per level; second, biclusters which are more coherent with the hierarchies *BP *and *MF *in Gene Ontology, according to the statistical test described in the Section "Construction and content". The second consequence is due to the constraint imposed on the interaction scores which allows HOCCLUS2 to focus on more reliable (functional) interactions, leading to the prediction of more functionally-coherent MGRNs.

After selecting the desired hierarchy, two types of queries can be performed: *i) *the retrieval of all the biclusters in the hierarchy, and *ii) *the exploration of only those biclusters containing miRNA(s) and/or gene(s) of interest. In the latter case, similarly to the "Search Interaction" function, a list of gene symbols and/or miRNAs IDs can be provided, both as single search criterion or in combination. In both cases, some filters can be applied, that is:

• on the specific levels of the hierarchy;

• on the p-values (*p_BP _*and *p_MF _*), measuring the biological significance of the genes in the biclusters;

• on the cohesiveness (computed through the compactness measure) of the biclusters, measuring the average strength of the interactions between miRNAs and genes in the biclusters;

• on the bicluster name.

The last filter actually lets ComiRNet return a single bicluster and is particularly useful to quickly retrieve and analyze biclusters that were considered interesting in a previous analysis. In this way, users can perform further investigations, for example through the graph-based visualization and the hierarchy browser.

**Figure 3:**
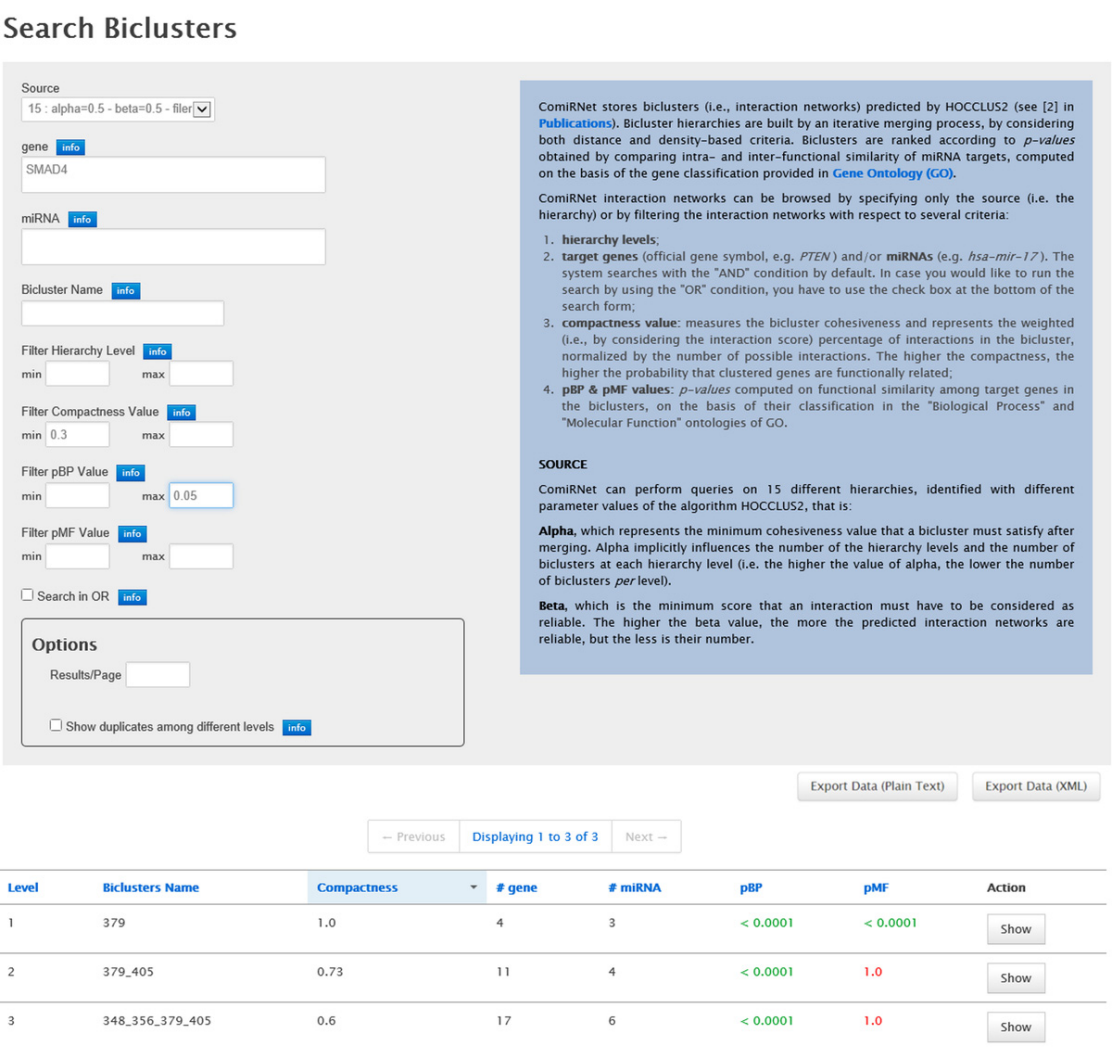
**ComiRNet MGRN search form**. Results obtained by searching for the MGRNs involving the gene SMAD4 in the hierarchy 15 (*α *= 0.5*, β *= 0.5), applying filters on the minimum compactness (0.3) and on the maximum *p_BP _*(0.05).

The results obtained from the query page include the list of all the biclusters matching the search criteria and parameter thresholds used. For each bicluster, ComiRNet reports the name/identifier, the hierarchy level, the compactness value, the *p_BP _*and *p_MF _*values and the number of genes and miRNAs involved. The results in the table can be dynamically sorted according to each column and can be exported as plain text or XML file. A complete view of the search form and of the query report is shown in Figure 3.

### Exploring and browsing MGRNs

**Figure 4:**
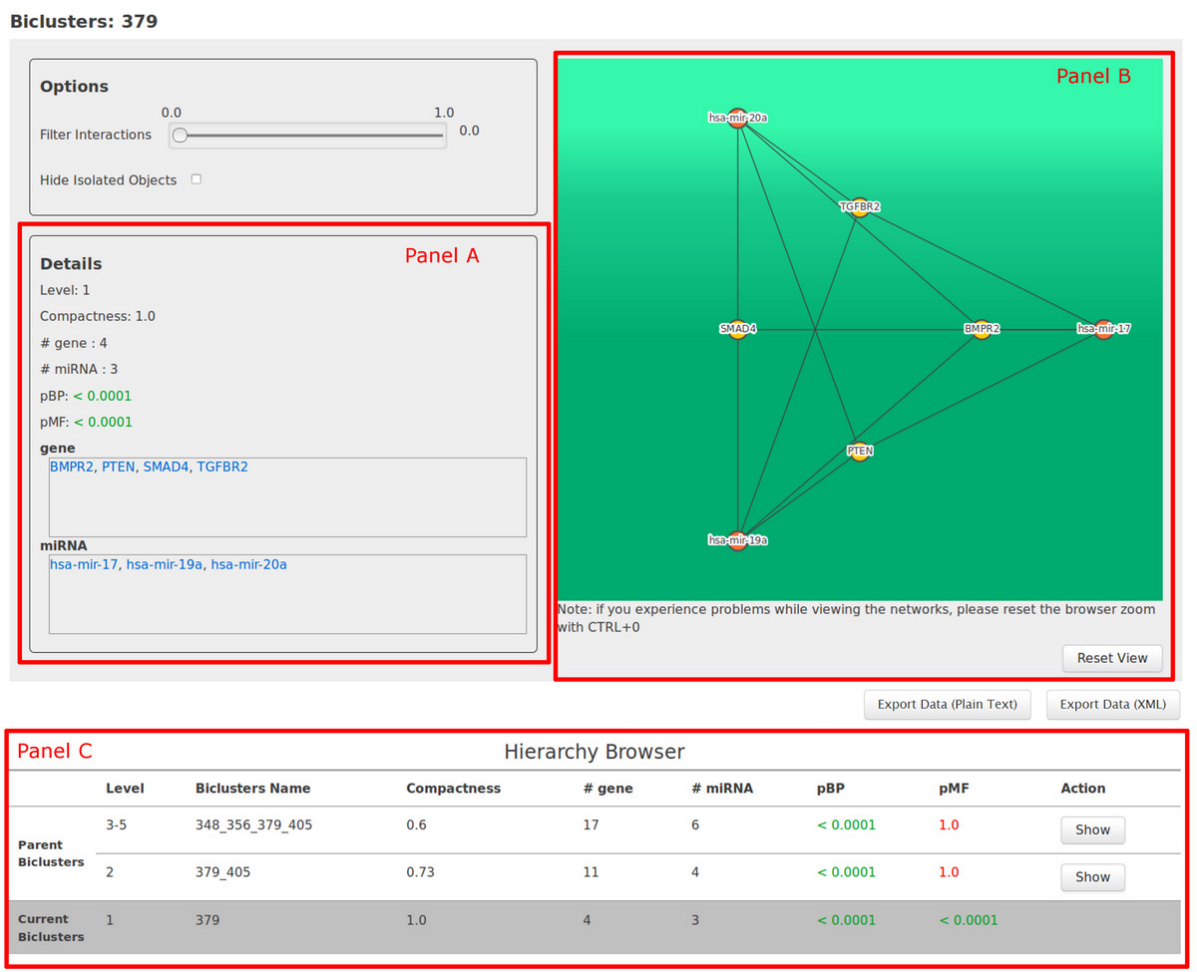
**MGRN details in ComiRNet**. Details of the bicluster × obtained by searching for the MGRNs involving the gene SMAD4 in the first level of the hierarchy 15 (*α *= 0.5*, β *= 0.5), applying filters on the minimum compactness (0.3) and on the maximum *p_BP _*(0.05).

Each retrieved bicluster can be analyzed through the "Show" button, which opens a new window (Figure 4) reporting the summary of the bicluster's properties (Figure 4, panel A), a dynamic graph-based visualization of the predicted miRNA-gene interactions network (Figure 4, panel B), and a comprehensive view of the bicluster hierarchy (i.e., parent and child biclusters) (Figure 4, panel C).

In the graph representing the interaction network, nodes represent miRNAs and target genes, whereas edges represent the miRNA-gene target interactions. When the user hovers the mouse pointer on a miRNA or on a gene, the system dynamically highlights all the predicted targets or all the miRNAs targeting the gene, respectively. This allows user awareness of the impact of a single miRNA on the whole set of genes involved in the bicluster or alternatively, of which are the miRNAs, among all those in the bicluster, that co-target a specific gene. This is particularly important when the user is exploring biclusters which do not belong to the first level of the hierarchy. Indeed, in this case, biclusters do not necessarily represent fully connected networks, and the identification of co-targeting entities becomes important. When the user hovers the mouse pointer on an edge, the system shows the predicted interaction score, enabling a quick evaluation of the reliability of a specific interaction, in the overall context of the network.

An additional function (called "Filter interactions" in the system), that further enables the analysis and the interpretation of the selected bicluster, consists in the possibility to set a threshold on the minimum score of interactions to be shown in the graph. To this aim, a slider allows the user to dynamically customize the network visualization by selecting the threshold value (in the interval 0 [1]). The system dynamically redraws the graph, excluding all those miRNA-gene interactions with a score below the selected threshold. Moreover, a check box allows the user to hide isolated nodes, i.e. miRNAs and genes that are not connected to any other nodes in the bicluster, according to the selected threshold. This option is particularly useful for an easier interpretation of predicted MGRNs in which a large number of miRNAs and genes is involved (e.g., biclusters belonging to high levels of the hierarchy). Indeed, by excluding weaker interactions it is possible to better highlight, among all the possible intra-bicluster connections, only the more strongly reliable ones. The application of this filter contextually modifies the list of miRNAs and genes belonging to the bicluster, reported in the summary of bicluster properties, thus facilitating the users in keeping (if they are interested) only those objects in the bicluster that are particularly interesting for further analysis.

All the functions provided by the dynamic visualization of biclusters represent, as a whole, a powerful tool to easily and quickly evaluate the biological consistency of a bicluster, by taking into account all its properties. This allows the user to include or discard a bicluster from the set of those that meet some specific requirements. Indeed, in ComiRNet, depending on the considered hierarchy, the same miRNA or gene may belong to different biclusters, each representing one of the possible interaction networks it can establish with other miRNAs and groups of target genes. Thus, even though a bicluster containing miRNAs and genes of interest has good quality parameters values (i.e. *p*-values and compactness), this does not necessarily mean that it is the one that best fits the specific analysis the user intends to pursue. On the contrary, other biclusters could be more informative, possibly belonging to different levels of the same hierarchy or to other hierarchies.

The "Hierarchy Browser" (Figure 4, panel C) allows the user to browse the hierarchy the bicluster belongs to, analyzing the details of its parent and child biclusters. Similarly to the interface used to show the results of queries on MGRNs, also in this case some information about listed biclusters is provided. Detailed properties of each bicluster can still be visualized by means of a specific function. The exploration of the biclusters in the hierarchy allows the user to uncover different functional relationships that miRNAs and genes can establish at different levels of granularity, and, thus, to interpret the biological phenomena in which they are involved at a system-wide level. This aspect is widely discussed in the next section.

## Discussion

ComiRNet provides unique opportunities for users, as compared to other similar tools, for an easy and quick searching and filtering of MTIs and MGRNs, at different granularity/specificity levels. In this section, we underline the main advantages of ComiRNet when compared to other commonly-used web-based systems which have been designed with similar aims. We also provide some examples which clarify how ComiRNet can support biologists and clinicians in the difficult task of managing and interpreting miRNA-gene target interaction data.

### Prediction of MTIs

Accuracy of miRNA-mRNA interaction predictions is fundamental in studies of miRNAs mechanisms and functions. However, a common problem that users face when they use miRNA prediction databases is, on the one hand, the huge amount of results they return (from hundreds to thousands of targets for only one miRNA) and, on the other hand, the uncertainty of such results [36,37].

ComiRNet is not intended to be comprehensive of all the aspects considered in the methodologies mentioned in the Section "Background", but it can be considered a valid approach for integrating and boosting their predictive capabilities as well as screening the huge (and often inconsistent) amount of data and detecting more reliable MTIs.

In order to show such advantages, we performed a quantitative analysis which compares ComiRNet with the databases extracted by 10 single prediction algorithms (i.e., DIANA-micro T, microCosm, miRanda, picTar 4-way, picTar 5-way, PITA All Targets, PITA Top Targets, TargetScan Conserved Target, TargetScan Non-Conserved Targets, RNA22 3' UTR) and with two web-based integrated resources, that is, mirDIP and TarMiR.

mirDIP stores predictions from 12 different databases and allows users to select the sources to consider for each query. However, when multiple sources are selected, mirDIP returns redundant results, making the identification of the most interesting and reliable interactions a very difficult task.

TarMiR 1.0 integrates pre-computed lists of miRNA targets from eight different data sources, concerning four different species. Similarly to mirDIP, TarMiR gives the opportunity to query for MTIs from one or more selected sources. Moreover, it can provide results as separate lists (one for each source) or as an integrated list. In the latter case, it is also possible to obtain a non-redundant list containing only target genes shared by all the selected sources. However, it does not provide any explanation of the criteria adopted to integrate redundant and/or contradictory predictions. In addition, interactions are not associated with a score (representing their reliability) and results can be filtered only by choosing a percentile threshold (cut-off) with respect to the total of MTIs retrieved. Obviously, the percentile threshold is applicable because TarMiR ranks MTIs. Ranking is based on the rankings returned by each source, that is, a single MTI is globally ranked in the top *p*% if it is ranked in the top *p*% in at least one source.

The result of the quantitative analysis is reported in Table 3, which shows the number of (ranked) interactions that each database must return in order to include a given percentage of validated interactions in miRTaRBase, when a query on two well-known miRNAs, i.e. hsa-mir-17 and hsa-mir-20a, is performed. Such analysis gives an idea of the noise (i.e. possible false positives) returned by a query performed on each database. Indeed, if a database which is asked to return a small set of validated interactions (existing in mirTarBase) returns a large amount of interactions, in order to include at least those originally required, it is probably returning many false positives.

**Table 3:** Number of target genes that must be returned by each database, in order to retrieve (cover) a given percentage of validated interactions coming from miRTarBase and involving hsa-mir-17 and hsa-mir-20a, respectively.

		Covered %							
**Database**	**No. Interactions**	**10%**	**20%**	**30%**	**40%**	**50%**	**60%**	**70%**	**AUPRC**

Diana-micro T	4106	359	1010	1999	-	-	-	-	0.0252
microCosm	1197	-	-	-	-	-	-	-	0.0010
picTar 4-way	0	-	-	-	-	-	-	-	0.0000
picTar 5-way	0	-	-	-	-	-	-	-	0.0000
PITA All Targets	8027	431	1373	2860	4710	6776	-	-	0.0233
PITA Top Targets	859	349	-	-	-	-	-	-	0.0157
TargetScan C	991	538	890	-	-	-	-	-	0.0135
TargetScan NC	2954	1250	2507	-	-	-	-	-	0.0057
Miranda	2336	920	1687	-	-	-	-	-	0.0087
RNA22 3' UTR	0	-	-	-	-	-	-	-	0.0000

mirDIP	20470	669	1813	3455	5653	9821	-	-	0.0173
TarMir	6759	564	1186	2562	4175	6464	-	-	0.0216
ComiRNet	9218	32	623	1647	4500	7854	-	-	**0.1664**

		**Covered %**							

**Database**	**No. Interactions**	**10%**	**20%**	**30%**	**40%**	**50%**	**60%**	**70%**	**AUPRC**

Diana-micro T	3857	326	798	1608	3789	-	-	-	0.0170
microCosm	1029	-	-	-	-	-	-	-	0.0008
picTar 4-way	610	278	-	-	-	-	-	-	0.0122
picTar 5-way	268	-	-	-	-	-	-	-	0.0040
PITA All Targets	8027	940	2095	3602	5035	6600	-	-	0.0090
PITA Top Targets	859	390	-	-	-	-	-	-	0.0087
TargetScan C	990	466	869	-	-	-	-	-	0.0083
TargetScan NC	2954	957	2389	-	-	-	-	-	0.0039
Miranda	2499	536	1410	-	-	-	-	-	0.0085
RNA22 3' UTR	1640	1153	-	-	-	-	-	-	0.0022

mirDIP	23873	493	1367	2861	5658	8755	12834	-	0.0130
TarMir	5963	427	1165	2108	3831	-	-	-	0.0142
ComiRNet	9879	18	599	1540	3983	6900	9524	-	**0.1624**

**Figure 5:**
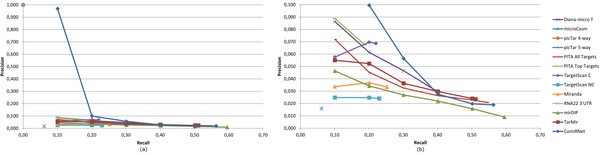
**Precision-Recall curves for hsa-mir-17a**. Precision-recall values are computed on interactions in miRTarBase: (a) Complete curves, (b) Curves after rescaling on the Precision axis (zoom-in).

**Figure 6:**
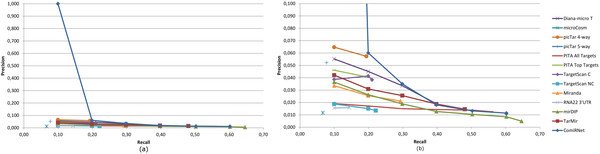
**Precision-Recall curves for hsa-mir-20**. Precision-recall values are computed on interactions in miRTarBase: (a) Complete curves, (b) Curves after rescaling on the Precision axis (zoom-in).

No. Interactions is the total number of interactions in a given database which involve the specified miRNA. The symbol "-" indicates that the database does not contain the specified percentage of interactions of miRTarBase. AUPRC is the area under the precision-recall curves (i.e. the area under the lines in Figures 5 and 6).

As can be observed from the table, ComiRNet is able to better filter out false positives, returning a smaller number of interactions with respect to other (both single and integrated) databases for most of the considered percentage of covered interactions. In some cases, these databases are not able to include the required percentage of validated interactions, returning the whole set of predictions involving the requested miRNA (such a situation is represented by the symbol "-"). There are few systems which, for some percentages of covered interactions, are able to return a smaller number of predictions. This is the case of TarMiR and Pita All Targets for hsa-mir-17 and TarMiR and Diana-micro T for hsa-mir-20a. However all these systems retrieve a smaller number of interactions only in few cases. An idea of the overall precision and recall capabilities of all the systems is provided by the last column of Table 3, which reports the AUPRC (Area Under the Precision-Recall Curve [38]). By analyzing AUPRC values, we can conclude that ComiRNet outperforms all the other approaches by a great margin. A detailed view of the precision-recall curves is provided in Figures 5 and 6.

Filtering out false positives, and, thus, improving the precision of the returned interactions, also allows researchers to focus their study on a small number of interactions which are worth being investigated. Moreover, differently from other integrated approaches, MTIs returned by ComiRNet are not redundant. This is due to the fact that ComiRNet considers the specific interaction to be predicted as a unit of analysis in a machine learning-based approach which exploits predictions of multiple algorithms, as well as validated interactions for training purposes. Finally, ComiRNet does not require a selection of the source databases and, thus, does not require any a-priori knowledge of the aspects of the methodologies adopted by prediction algorithms. This feature significantly helps biologists and clinicians who do not have a deep knowledge of the best approach to consider. Indeed, the choice of a specific prediction algorithm can significantly change the query response. Assessing which one is the best for the specific needs of the user is not a trivial task.

As already stated in the Section "Utility", ComiRNet can also be exploited to assess the co-occurrence of multiple miRNAs targeting in one (or more) gene(s). However, searching for MTIs which involve either multiple miRNAs or multiple genes is not the same as browsing MGRNs, since searching is based on a combinatorial analysis performed directly on the set of MTIs and not on the set of (possibly overlapping) regulatory networks returned by HOCCLUS2. Among the considered competitors, only mirDIP provides this function, but, in this case, the results are still redundant. Among other systems, miRTar provides such a function, but the users have to know in advance the specific interactions they are searching for (i.e., the miRNA(s) and the gene(s) involved in the interaction(s)).

Other tools make predictions on the basis of expression data and are not comparable with ComiRNet. Indeed, active concurrency in expression profile studies are not considered by ComiRNet, because they introduce a bias related to qualitative and quantitative properties consistent with the specific context in which the system is analyzed. As already stated, this is motivated by the fact that ComiRNet has been developed to widely explore, without any a-priori knowledge, any possible biological scenarios.

### Searching MGRNs

The effectiveness of our method for the study of miRNA functions and mechanisms, in the context of MGRNs, has already been discussed in our previous work [24,25]. In particular, many examples were provided by considering, as a case study, the miR-17-92 gene cluster and its paralogs, with the support of what was reported in several papers found in the literature. With the development of ComiRNet, the underlying information implicitly available in the predicted data can now be easily exploited by any user.

To the best of our knowledge, other web-based systems which work on MGRNs cannot be directly compared with ComiRNet because they are based on different methodologies and use different types of data (e.g., gene expression data). In this subsection, we provide some examples which illustrate the main advantages of ComiRNet in the investigation of miRNAs functional properties, by means of the study of MGRNs stored in the database.

As already discussed in the Section "Utility", MGRNs stored in ComiRNet are identified by the algorithm HOCCLUS2, which requires the parameters *α *and *β*. Since the combination of these parameters influences the obtained hierarchy of biclusters (see Table 2), several hierarchies can be analyzed in order to have a complete picture of all the possible scenarios. For example, some miRNAs and genes might not be involved in any MGRN, belonging to a given hierarchy, although they are involved in some MTIs. This is due to the fact that HOCCLUS2 discards objects that cannot fall in any bicluster, according to some statistics computed from data which mainly depend on the parameter *β *(see Section "Construction and content" for details). An example is SMAD4, a central cellular transducer of TGF-*β *signaling (Transforming Growth Factor *β *signaling) which plays pivotal roles in a variety of biological processes [39]. Among the 15 hierarchies stored in ComiRNet, only those extracted with *β *= 0.5, i.e. those with ID 3, 6, 9, 12 and 15, contain biclusters including SMAD4. Thus, if a user queries ComiRNet for SMAD4, by randomly choosing a hierarchy, it is possible that the system will not return any result.

**Figure 7:**
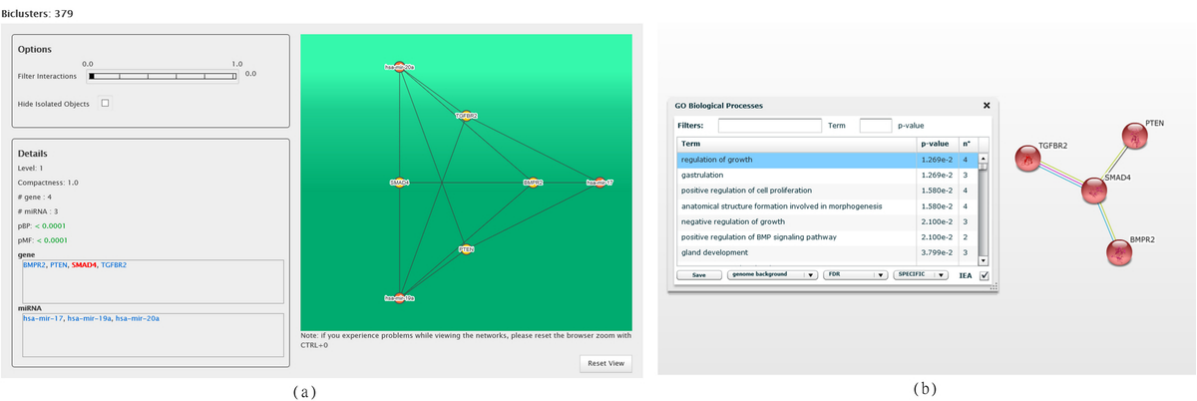
**Bicluster × in ComiRNet (a) and the enrichment of its genes in STRING (b)**. The represented bicluster × is obtained by searching for the MGRNs involving the gene SMAD4 in the first level of the hierarchy 15 (*α *= 0.5*, β *= 0.5), applying filters on the minimum compactness (0.3) and on the maximum *p_BP _*(0.05). In STRING, red nodes are those involved in the category (i.e., biological process) selected in the enrichment analysis table.

**Figure 8:**
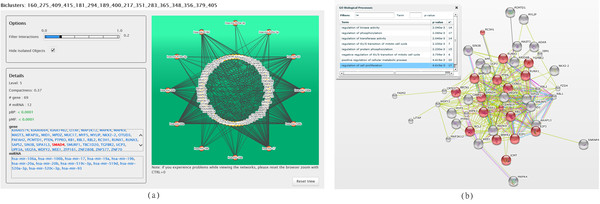
**Bicluster Y in ComiRNet (a) and the enrichment of its genes in STRING (b)**. The represented bicluster Y (shown with the filter on the interaction score set to 0.2), is obtained by searching for the MGRNs involving the gene SMAD4 in the fifth level of the hierarchy 3 (*α *= 0.1*, β *= 0.5), applying filters on the minimum compactness (0.3) and on the maximum *p_BP _*(0.05). In STRING, red nodes are those involved in the category (i.e., biological process) selected in the enrichment analysis table.

**Figure 9:**
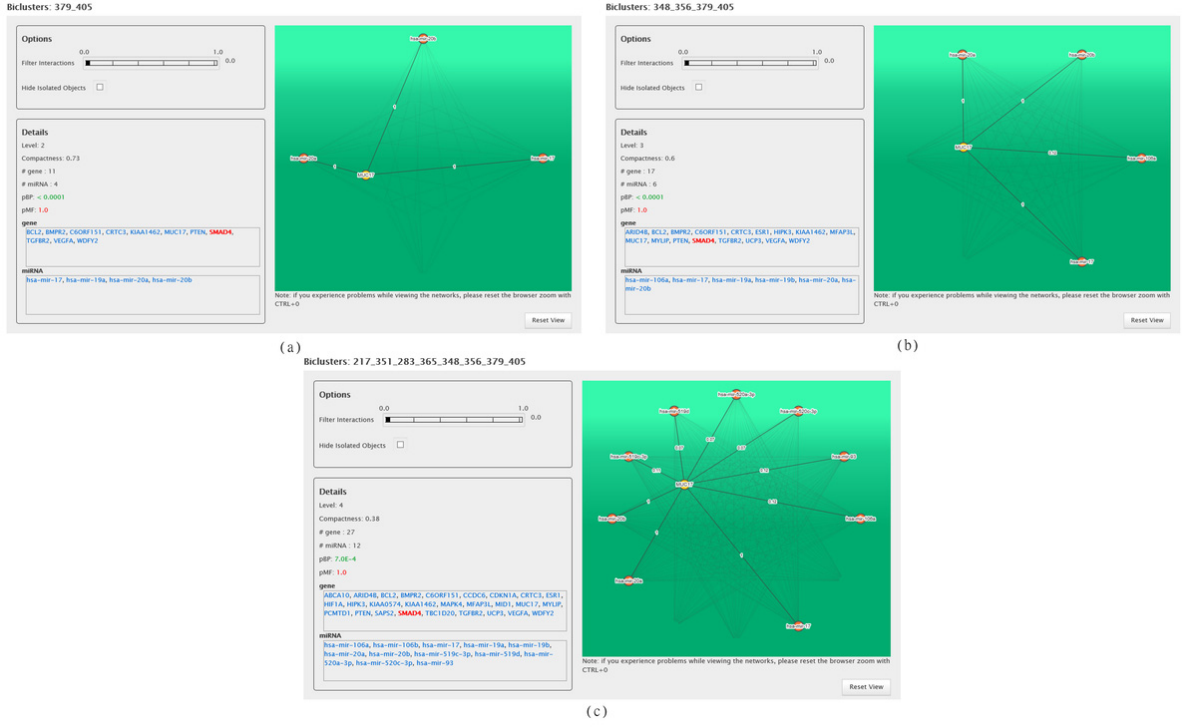
**Biclusters Z' (a), Z" (b) and Z"' (c) in ComiRNet**. The represented biclusters are obtained by searching for the MGRNs involving the gene SMAD4 in the hierarchy 3 (*α *= 0.1*, β *= 0.5), applying filters on the minimum compactness (0.3) and on the maximum *p_BP _*(0.05). Biclusters Z' (a), Z" (b) and Z"' (c) belong to the second, third and fourth level of the considered hierarchy, respectively.

The main differences among the hierarchies is the number of levels and their significance, ranging from eight levels (7 with *p_BP _≤ *0.05) in hierarchy 3, to three levels in hierarchy 15 (all highly statistically significant). The picture that the user obtains by analyzing hierarchy 3 and hierarchy 15 is quite different, but both the scenarios are worth to be investigated. The former, even though it includes MGRNs based on weak MTIs, provides a much larger spectrum of the diverse possibilities that are essential for the discovery of miRNAs and genes that can be central in inter-pathway connections and in the regulations of biological processes. The latter can help to immediately detect, among all the networks predicted, the most strongly reliable ones. These situations represent the borderline cases of a wide scale of analysis scenarios that biclusters identified in intermediate hierarchies can depict. Figures 7, 8 and 9 help to clarify these concepts and to illustrate how ComiRNet is essential to facilitate the users in performing these types of analyses.

In particular, Figure 7(a) shows the graph-based representation of the bicluster 379 (henceforth referred to as bicluster X), which is the smallest bicluster belonging to the first level among all the hierarchies. In Figure 8(a) we report the bicluster 160_275_409_415_181_294_189_400_217_351_283_365_348_356_379_405 (henceforth referred to as bicluster Y), which is one of the largest biclusters. Bicluster Y is extracted in hierarchies 3 and 6, but not in hierarchies 9, 12 and 15. In Figures 7(b) and 8(b) we show the functional analysis in STRING [40] of the genes grouped in biclusters × and Y, respectively.

STRING is a protein-protein interaction (PPI) database, which stores interaction networks based on known and predicted PPIs. It provides tools for the statistical enrichment of network components according to GO and to three databases of pathways. The functional analysis using STRING is performed by providing it the list of genes involved in the interaction networks extracted by ComiRNet. The combination of ComiRNet and STRING can significantly improve the interpretation of MGRNs, by helping users to form many functional and likely biologically coherent hypotheses on miRNAs and genes involved in the biclusters. In particular, this combination can support the detection of functional relationships between miRNAs and genes involved in ComiRNet biclusters, in the context of specific biological processes, supported by direct and indirect PPIs.

In Figures 7(b) and 8(b) we show the biological processes (see the table on the left side of the screenshot), in which genes grouped in the considered biclusters appear to be significantly enriched. Actually, the biological processes selected and shown in the pictures are only a few of those returned by STRING, each reflecting known functional properties of the miRNAs included in the two considered biclusters.

In the considered cases, genes included in the biclusters are strictly related to each other (PPI network confidence value in STRING: *p*-value = 0). In particular, genes in bicluster × may be the central nodes from which a cascade of biological events can be triggered or deactivated, depending on the genes and miRNAs that are considered in bicluster Y. Indeed, bicluster × includes key genes (i.e., TGFBR2, BMPR2, SMAD and PTEN) of two related, crucial signaling pathways with antagonistic functions: the TGF-*β *signaling (tumour suppression) and the phosphatidylinositol 3-kinase (PI3K)/protein kinase B (PKB/Akt) signaling (oncogenic unction). The tumour-suppressive effects of TGF-*β *signaling are largely due to its ability to inhibit cell proliferation and trigger apoptosis. Akt promotes cell proliferation, growth and cell survival through multiple complementary downstream pathways [41]. PTEN (phosphatase and tensin homologue) is a tumor suppressor that negatively regulates cell survival and proliferation by antagonizing the PKB/Akt signaling [42]. The analysis in STRING indicates that SMAD4 and PTEN are two co-expressed genes. Moreover, evidence of their synergistic action in the negative regulation of cell proliferation and tumorogenesis are reported in the literature [43]. This evidence strongly supports the coordinated control of these two genes by the same miRNAs, as suggested by the MGRN identified by ComiRNet.

In order to clarify the functional inter-relationships of these genes with those belonging to bicluster Y, a similar analysis has to be carried out on biclusters at intermediate levels of the hierarchy that contributed to the definition of this bicluster from lower levels. This analysis is essential to obtain an insight on how miRNAs involved in biclusters at different levels of the hierarchy can modulate, by means of alternative and synergistic cooperation, diverse related pathways that then lead to the definition of the biclusters at the higher level. This analysis could also be pivotal for the discovery of new functions of both miRNAs and genes. To better illustrate this concept, we use as an example the case of the MUC17 gene, coding for a membrane mucin. Mucins are high molecular weight glycoproteins that play important roles in carcinogenesis and tumor invasion. MUC17 is a membrane-associated protein that is mainly expressed in the digestive tract. The physiological function of MUC17 is still unclear. It has been suggested that MUC17 may conduct signals in response to external stimuli that lead to cellular responses, including proliferation, differentiation, apoptosis or secretion of cellular products. A recent study correlates its expression with the malignancy potential of pancreatic ductal adenocarcinomas (PDACs) [44]. According to this study, this gene is a validated target of miR-17, miR-20a and miR-20b, that is, exactly what ComiRNet predicts in the bicluster 379 405 (henceforth referred to as bicluster Z'), as shown in Figure 9(a). This bicluster is identified in all the hierarchies with *β *= 0.5 and contributes to the definition of bicluster Y at a higher level of the hierarchy. This suggests that MUC17 may be in some way related with some of the genes of this bicluster and that its miRNA-dependent expression may be much more complex that the one envisaged by the study in [44].

Figures 9(a), 9(b) and 9(c) show the graph-based visualization of biclusters containing MUC17 at levels 2, 3 and 4 of the hierarchy, respectively. miRNAs that may cooperatively target this gene are highlighted, together with the prediction score assigned to each specific MTI. We can see that, while bicluster Z' associates MUC17 with miR-17, miR-20a and miR-20b (Figure 9(a)), biclusters at levels 3 and 4 (Figures 9(b) and 9(c)) also include other miRNAs, some belonging to the miR-17-92 gene cluster and others to the miR-520 gene cluster. Reconstructing miRNA targeting on MUC17 and analyzing functions of genes belonging to these biclusters may help, not only to discover other miRNAs which could concur to its regulation, and hence to its aberrant expression in cancer pathogenesis, but also to give an insight, through the putative relationships suggested by the biclustering with other genes, on the still unknown physiological role of MUC17.

## Conclusions

ComiRNet is a unique web-based resource specifically developed to provide an easy access to MTIs and MGRNs, predicted by a combined data-mining approach, which aims to support the study of miRNA functions and roles in complex biological processes. As for the prediction of MTIs, ComiRnet provides unique advantages with respect to similar systems, since users can retrieve more reliable (i.e. with a low amount of false positives and false negatives) and non-redundant predictions, without any a-priori knowledge on the specific features considered by the combined prediction algorithms. This advantage is due to the adopted semisupervised ensemble-based learning approach, which learns to combine predictions from ten different algorithms, also exploiting experimentally validated data.

As for MGRNs, we showed that genes in a bicluster are likely to function together as a network and that miRNAs in the same bicluster are likely to cooperatively target groups of networked genes. The identification of MGRNs, based on the biclustering algorithm HOCCLUS2, does not introduce any bias possibly due to context-specific features of miRNAs or possibly due to the presence of the same object in more than one network. Moreover, the hierarchical organization of biclusters improves the interpretability of the results and emphasizes similarities among genes at different granularity levels, allowing ComiRNet users to explore many possible biological scenarios. It is also noteworthy that the functional relationships suggested by miRNAs and target genes in biclusters can help to detect unknown functional similarities or synergies among miRNAs and among target genes, that can enable the discovery of new miRNA and gene functions.

The ComiRNet web interface is easy to use, is equipped with a powerful, flexible query system and provides a user-friendly GUI to browse hierarchies and to graphically visualize MRGNs. Moreover, ComiRNet allows users to extend the analysis of each miRNA, gene and MTI stored in the database, through hyperlinks to several external resources. Although ComiRNet cannot provide a unique solution to all the many challenging tasks regarding the research on miRNAs, it represents a useful resource for the miRNA research community. This includes the discovery of new miRNA functions and regulatory mechanisms, through the analysis of the complex interactions that they can establish in regulatory networks.

Future plans for further developments include the integration of data on pathways and PPI networks, in order to obtain a more comprehensive view of the biological processes in which miRNAs and target genes can be involved. This is in line with recent research on gene function classification when PPI network data can be exploited [45].

## Availability and requirements

ComiRNet can be accessed at: http://comirnet.di.uniba.it.

The semi-supervised miRNA target prediction system can be downloaded at the following hyperlink: http://www.di.uniba.it/~ceci/micFiles/systems/semisupervised_HOCCLUS2/.

HOCCLUS2 can be downloaded at the following hyperlink: http://www.di.uniba.it/~ceci/micFiles/systems/HOCCLUS/.

## Competing interests

The authors declare that they have no competing interests.

## Authors' contributions

MC and GP contributed to the definition of the methods. DD contributed to the conception of the biological investigation and of the database. GP and MC contributed to the design of the database and of the web-based system. GP and DD took care of the review and selection of bioinformatic resources. GP implemented the system and ran the experiments. DD performed the biological analysis and validation of the results. GP and MC performed the analysis of the results, from the computer science point of view. MC, GP and DD contributed to the manuscript drafting. MC, GP, DD and DM contributed to the manuscript finalization. DM and MC supervised the study. All the authors read and approved the final manuscript.
